# Supplementary material for: Epithelial Ca2+ waves triggered by enteric neurons heal the gut
Source: bioRxiv. 2023 Aug 15:2023.08.14.553227. Preprint. [Version 1] doi: 10.1101/2023.08.14.553227 (PMC10461974; doi:10.1101/2023.08.14.553227)
Supplement: Supplement 1 [file NIHPP2023.08.14.553227v1-supplement-1.pdf]

bioRxiv preprint doi: https://doi.org/10.1101/2023.08.14.553227; this version posted August 15, 2023. The copyright holder for this preprint (which was not certified by peer review) is the author/funder, who has granted bioRxiv a license to display the preprint in perpetuity. It is made available under aCC-BY 4.0 International license.

Supplementary Information

Supplementary Tables

S. Table 1

Average expression, fold change and p-value of the 20 most significantly upregulated genes in EC clusters during Homeostasis as shown in Figure 1E.

S. Table 1: Top 20 upregulated genes in EC clusters during Homeostasis

Feature ID	Feature Name	Homeostasis Average	Homeostasis Log2 Fold Change	Homeostasis P-Value	Recovery d2 Average	Recovery d2 Log2 Fold Change	Recovery d2 P-Value
FBgn0032889	CG9331	2.226547128	1.289501629	6.07191E-05	0.910650724	-1.289501629	6.07191E-05
FBgn0259236	comm3	1.228206322	1.228570472	0.000348801	0.523863811	-1.228570472	0.000348801
FBgn0038755	Hs6st	2.471985271	1.199742456	0.000507757	1.076005724	-1.199742456	0.000507757
FBgn0034808	CG9896	2.06405015	1.169602911	0.000560433	0.917360782	-1.169602911	0.000560433
FBgn0262733	Src64B	1.770201449	1.084677475	0.001604504	0.834443637	-1.084677475	0.001604504
FBgn0038194	Cyp6d5	1.245471626	1.043748644	0.002846527	0.603905217	-1.043748644	0.002846527
FBgn0036767	CG16775	2.705574676	0.940550996	0.009212188	1.409591463	-0.940550996	0.009212188
FBgn0033188	Drat	1.270523243	0.936112546	0.011384258	0.663816449	-0.936112546	0.011384258
FBgn0026575	hang	2.10501293	0.924281577	0.012369538	1.109076724	-0.924281577	0.012369538
FBgn0031580	CG15423	1.664578414	0.933537345	0.01266869	0.871348956	-0.933537345	0.01266869
FBgn0030098	CG12057	1.875147414	0.920260944	0.024184345	0.99069213	-0.920260944	0.024184345
FBgn0031011	CG8034	2.73231897	0.839338308	0.028253279	1.527017478	-0.839338308	0.028253279
FBgn0085358	Diedel3	5.826532251	0.834393808	0.02918147	3.267798231	-0.834393808	0.02918147
**FBgn0000024**	**Ace**	**1.063678132**	**0.827671636**	**0.036466398**	**0.599112319**	**-0.827671636**	**0.036466398**
FBgn0043364	cbt	1.549814923	0.804559619	0.037446637	0.887165522	-0.804559619	0.037446637
FBgn0001145	Gs2	1.901214637	0.82620092	0.040936694	1.072171406	-0.82620092	0.040936694
FBgn0033853	CG6145	1.379531632	0.790243027	0.042427252	0.797538319	-0.790243027	0.042427252
FBgn0051431	CG31431	1.003080301	0.794730924	0.043818809	0.578023565	-0.794730924	0.043818809
FBgn0025574	Pli	1.99532747	0.728692295	0.079581142	1.203976116	-0.728692295	0.079581142
FBgn0003748	Treh	1.237008242	0.722319902	0.084101708	0.749609333	-0.722319902	0.084101708

bioRxiv preprint doi: https://doi.org/10.1101/2023.08.14.553227; this version posted August 15, 2023. The copyright holder for this preprint (which was not certified by peer review) is the author/funder, who has granted bioRxiv a license to display the preprint in perpetuity. It is made available under aCC-BY 4.0 International license.

S. Table 2

Average expression, fold change and p-value of the 20 most significantly upregulated genes in EC clusters during Recovery d2 as shown in Figure 1E.

S. Table 2: Top 20 upregulated genes in EC clusters during Recovery d2

Feature ID	Feature Name	Homeostasis Average	Homeostasis Log2 Fold Change	Homeostasis P-Value	Recovery d2 Average	Recovery d2 Log2 Fold Change	Recovery d2 P-Value
FBgn0267910	lncRNA:CR34335	15.237477	-2.112901065	2.67957E-15	65.92152665	2.112901065	2.67957E-15
FBgn0263005	CG43313	0.888655346	-1.780268198	1.53628E-10	3.053555666	1.780268198	1.53628E-10
FBgn0034709	Swim	0.716002307	-1.498659054	5.29143E-06	2.024041058	1.498659054	5.29143E-06
FBgn0265991	Zasp52	1.879209838	-1.361805013	8.56648E-06	4.830762448	1.361805013	8.56648E-06
FBgn0041184	Socs36E	1.573173864	-1.307146591	1.52612E-05	3.893750782	1.307146591	1.52612E-05
FBgn0033448	hebe	1.138155913	-1.205815961	8.82001E-05	2.626029115	1.205815961	8.82001E-05
FBgn0052982	CG32982	1.54270568	-1.193673448	0.00018319	3.529490492	1.193673448	0.00018319
FBgn0040687	CG14645	1.477368354	-1.138606279	0.000277664	3.253419535	1.138606279	0.000277664
FBgn0004456	mew	1.16253046	-1.149757158	0.000342608	2.580017289	1.149757158	0.000342608
FBgn0034289	CG10910	2.298655161	-1.134609312	0.000691557	5.047880752	1.134609312	0.000691557
FBgn0085249	CG34220	0.597853463	-1.060262119	0.001593676	1.247112203	1.060262119	0.001593676
FBgn0085353	CG34324	0.783709381	-1.03348397	0.002067007	1.604662434	1.03348397	0.002067007
FBgn0029762	NAAT1	0.733606147	-1.065348447	0.00386101	1.535644695	1.065348447	0.00386101
FBgn0027932	Akap200	0.85615595	-0.97084245	0.00386101	1.678473072	0.97084245	0.00386101
FBgn0001186	Hex-A	0.951284389	-0.951035954	0.004391881	1.839514463	0.951035954	0.004391881
FBgn0001104	Galphai	1.117505256	-0.859047413	0.012938004	2.027396086	0.859047413	0.012938004
FBgn0011661	Moe	5.50119976	-0.821700318	0.01903966	9.724791157	0.821700318	0.01903966
FBgn0031170	ABCA	0.574494523	-0.853251542	0.023203517	1.038141826	0.853251542	0.023203517
FBgn0267698	Pak	1.599579623	-0.804512541	0.023988656	2.794259854	0.804512541	0.023988656
FBgn0027556	CG4928	2.73231897	-0.852334597	0.025952859	4.933809767	0.852334597	0.025952859

bioRxiv preprint doi: https://doi.org/10.1101/2023.08.14.553227; this version posted August 15, 2023. The copyright holder for this preprint (which was not certified by peer review) is the author/funder, who has granted bioRxiv a license to display the preprint in perpetuity. It is made available under aCC-BY 4.0 International license.

Supplementary Movie Legends

Movies S1-S3: Movies depicting cholinergic sensitivity assay described on Fig. 1I. **S1.** *mex*^{TS} > *GCaMP7c* midgut during Homeostasis. **S2.** *mex*^{TS} > *GCaMP7c* midgut during Recovery d2. **S3.** *mex*^{TS} > *GCaMP7c* + *dCas9VPR, gRNA-Ace* gut during Recovery d2.

Movies S4-S6: Movies depicting nicotinic sensitivity assay described on Fig. 2F-G. **S6.** *mex*^{TS} > *GCaMP7c* midgut during Homeostasis. **S7.** *mex*^{TS} > *GCaMP7c* midgut during Recovery d2. **S8.** *mex*^{TS} > *GCaMP7c* + *nAChRβ3*^{RNAi} gut during Recovery d2.

Movie S7: Movie of R49E06 innervations at R4 in R49E06^{TS}> *syt1HA* + *mexLexA>6xLexAopGFP* fly during Recovery d2 as described on Fig. 4D

Movies S8-S9: Movies depicting nicotine and heptanol assay during Recovery d2 as described on Fig. 5E. **S8** midgut from *mexLexA* > *LexAopGCaMP7c* flies administered Nicotine. **S9.** midgut from *mexLexA* > *LexAopGCaMP7c* flies administered nicotine and heptanol.

Full Genotypes

Fig. 1:

Ore R corresponds to *Oregon R*

Myo1A^{TS}> *dCas9VPR* corresponds to +*; Myo1A-Gal4 Tubulin-Gal80*^{TS}*/+; UAS-3XFLAG-dCas9-VPR/+*

Myo1A^{TS}> *dCas9VPR, gRNA-ACE* corresponds to +*; Myo1A-Gal4 Tubulin-Gal80*^{TS}*/gRNA-ACE; UAS-3XFLAG-dCas9VPR/+*

Mex^{TS}*>GCAMP7c* corresponds to +*; Mex-Gal4 Tubulin-Gal80*^{TS}*/+; 20XUAS-IVS-jGCaMP7c/+*

Mex^{TS}*>GCAMP7c* + *dCas9VPR, gRNA-ACE* corresponds to +*; Mex-Gal4 Tubulin-Gal80*^{TS}*/gRNA-ACE; 20XUAS-IVS-jGCaMP7c/UAS-3XFLAG-dCas9-VPR*

Fig. 2:

Myo1A^{TS} > + corresponds to +*; Myo1A-Gal4 Tubulin-Gal80*^{TS}*/+; UAS- Luciferase*^{RNAi}*/+*

Myo1A^{TS} > *nAChRβ3*^{RNAi} corresponds to +*; Myo1A-Gal4 Tubulin-Gal80*^{TS}*/+; UAS-nAChRβ3*^{RNAi}*/+*

Mex^{TS} > + corresponds to +*; Mex-Gal4 Tubulin-Gal80*^{TS}*/UAS- emptyVK37; +*

Mex^{TS} > *nAChRβ3*^{RNAi} corresponds to +*; Mex-Gal4 Tubulin-Gal80*^{TS}*/+; UAS-nAChRβ3*^{RNAi}*/+*

bioRxiv preprint doi: https://doi.org/10.1101/2023.08.14.553227; this version posted August 15, 2023. The copyright holder for this preprint (which was not certified by peer review) is the author/funder, who has granted bioRxiv a license to display the preprint in perpetuity. It is made available under aCC-BY 4.0 International license.

Mex^{TS} > + / esg-GFP corresponds to +*; Mex-Gal4 Tubulin-Gal80^{TS}/esg-GFP; +*

Mex^{TS} > nAChRβ3^{RNAi}/ esg-GFP corresponds to +*; Mex-Gal4 Tubulin-Gal80^{TS}/esg-GFP; UAS-nAChRβ3^{RNAi}/+*

Mex > GFP / nAChRβ3-flag corresponds to +*; Mex-Gal4 UAS-2x-GFP/nAChRβ3-flag; +*

Myo1A > GFP / nAChRβ3-flag corresponds to +; *Myo1A-Gal4 UAS-GFP/nAChRβ3-flag; +*

Mex^{TS}>GCAMP7c corresponds to +*; Mex-Gal4 Tubulin-Gal80^{TS}/+; 20XUAS-IVS-jGCaMP7c/+*

Mex^{TS}>GCAMP7c + nAcRβ3^{RNAi} corresponds to +*; Mex-Gal4 Tubulin-Gal80^{TS}/+; 20XUAS-IVS-jGCaMP7c/UAS-nAcRβ3^{RNAi}*

Fig. 3:

Myo1A^{TS} > + corresponds to +*; Myo1A-Gal4 Tubulin-Gal80^{TS}; UAS-Luciferase^{RNAi}* and +*; Myo1-Gal4 Tubulin-Gal80^{TS}/UAS-2x-GFP; +*

Myo1A^{TS} > nAChRβ3^{RNAi} corresponds to +*; Myo1A-Gal4 Tubulin-Gal80^{TS}/+; UAS-nAChRβ3^{RNAi}/+*

Mex^{TS} > + corresponds to +*; Mex-Gal4 Tubulin-Gal80^{TS}/UAS- emptyVK37; +*

Mex^{TS} > nAChRβ3^{RNAi} corresponds to +*; Mex-Gal4 Tubulin-Gal80^{TS}/+; UAS-nAChRβ3^{RNAi}/+*

Myo1A^{TS} > NFAT-CaLexA corresponds to +*; Myo1A-Gal4 Tubulin-Gal80^{TS}/LexAop-CD8-GFP-2A-CD8GFP; UAS-mLexA-VP16-NFAT LexAop-rCD2-GFP/+*

Mex^{TS} > NFAT-CaLexA corresponds to +*; Mex-Gal4 Tubulin-Gal80^{TS}/LexAop-CD8-GFP-2A-CD8GFP; UAS-mLexA-VP16-NFAT LexAop-rCD2-GFP/ +*

Mex^{TS} > NFAT-CaLexA + nAChRβ3^{RNAi} corresponds to +*; Mex-Gal4 Tubulin-Gal80^{TS}/LexAop-CD8-GFP-2A-CD8GFP; UAS-mLexA-VP16-NFAT LexAop-rCD2-GFP/UAS-nAcRβ3^{RNAi}*

Myo1A^{TS} > + corresponds to +*; Myo1A-Gal4 Tubulin-Gal80^{TS}/UAS-emptyVK37; +/+*

Myo1A^{TS} > nAChRβ3^{RNAi-2} corresponds to +*; Myo1A-Gal4 Tubulin-Gal80^{TS}/UAS-nAChRβ3^{RNAi-2}; UAS-Luciferase/+*

Myo1A^{TS} > Orai corresponds to +*; Myo1A-Gal4 Tubulin-Gal80^{TS}/UAS-emptyVK37; UAS-Orai / +*

Myo1A^{TS} > nAChRβ3^{RNAi-2} + Orai corresponds to +*; Myo1A-Gal4 Tubulin-Gal80^{TS}/UAS-nAChRβ3^{RNAi-2}; UAS-Orai /+*

Mex^{TS} > nAChRβ3 corresponds to +*; Mex-Gal4 Tubulin-Gal80^{TS}/UAS-nAcRβ3;* +

Myo1A^{TS} > nAChRβ3 corresponds to +*; Myo1A-Gal4 Tubulin-Gal80^{TS}/UAS-nAcRβ3;* +

Fig. 4:

R49E06 > 6xmCherry corresponds to +; +; *R49E06-Gal4/20xUAS-6xmCherry-HA*

bioRxiv preprint doi: https://doi.org/10.1101/2023.08.14.553227; this version posted August 15, 2023. The copyright holder for this preprint (which was not certified by peer review) is the author/funder, who has granted bioRxiv a license to display the preprint in perpetuity. It is made available under aCC-BY 4.0 International license.

R49E06^{TS}*> myrGFP+syt1HA* corresponds to +; *Tubulin-Gal80*^{TS}*/10xUAS-IVS-myr::GFP; R49E06-Gal4/5xUAS-IVS-Syt1::smGdP-HA*

R49E06^{TS}*> syt1HA + mexLexA > LexAop6xGFP* corresponds to +; *Tubulin-Gal80*^{TS}*/ 13xLexAop2-6xGFP; R49E06-Gal4 mex-LexA::GAD/ 5xUAS-IVS-Syt1::smGdP-HA*

R49E06^{TS}*>* + corresponds to +; *Tubulin-Gal80*^{TS}*/+; R49E06-Gal4; UAS-Luciferase*^{RNAi}

R49E06^{TS}*> ChAT*^{RNAi} corresponds to +*; Tubulin-Gal80*^{TS}*/+; R49E06-Gal4; UAS-ChAT*^{RNAi}

ARCENs + mexLexA^{TS} > + corresponds to +*; Tubulin-Gal80*^{TS}*/ UAS-emptyVK37; R49E06-Gal4 mex-LexA::GAD / +*

ARCENs> shibire^{TS} corresponds to +*; +; R49E06-Gal4/UAS-shibire*^{TS}

mexLexA^{TS} *> LexAopnAChR*β*3* corresponds to +*; Tubulin-Gal80*^{TS}*/LexAop-nAChR*β*3*; *mex-LexA::GAD/+*

ARCENs > shibire^{TS} + *mexLexA*^{TS}*> LexAopnAChR*β*3* corresponds to +*; Tubulin-Gal80*^{TS}*/ LexAop-nAChR*β*3*; *R49E06-Gal4 mex-LexA::GAD / UAS-shibire*^{TS}

QUASCsChrimson + *Mex* ^{TS} > *NFAT-CaLexA* corresponds to *QUASCsChrimson/+; Mex-Gal4 Tubulin-Gal80*^{TS}*/LexAop-CD8-GFP-2A-CD8GFP; UAS-mLexA-VP16-NFAT LexAop-rCD2-GFP/+*

ARCENsQF > *QUASCsChrimson* + *Mex*^{TS} > *NFAT-CaLexA* corresponds to *QUASCsChrimson/+; Mex-Gal4 Tubulin-Gal80*^{TS}*/LexAop-CD8-GFP-2A-CD8GFP; UAS-mLexA-VP16-NFAT LexAop-rCD2-GFP/R49E06-QF*

Fig. 5:

Mex^{TS} > *NFAT-CaLexA* corresponds to +*; Mex-Gal4 Tubulin-Gal80*^{TS}*/ LexAop-CD8-GFP-2A-CD8GFP; UAS-mLexA-VP16-NFAT LexAop-rCD2-GFP/ +*

Mex^{TS} > *NFAT-CaLexA + nAChR*β*3*^{RNAi} corresponds to +*; Mex-Gal4 Tubulin-Gal80*^{TS}*/ LexAop-CD8-GFP-2A-CD8GFP; UAS-mLexA-VP16-NFAT LexAop-rCD2-GFP/ UAS-nAChR*β*3*^{RNAi}

Mex^{TS} > *NFAT-CaLexA + inx2*^{RNAi} corresponds to +*; Mex-Gal4 Tubulin-Gal80*^{TS}*/ LexAop-CD8-GFP-2A-CD8GFP; UAS-mLexA-VP16-NFAT LexAop-rCD2-GFP/UAS-inx2*^{RNAi}

Mex^{TS} > *NFAT-CaLexA + inx7*^{RNAi} corresponds to +*; Mex-Gal4 Tubulin-Gal80*^{TS}*/ LexAop-CD8-GFP-2A-CD8GFP; UAS-mLexA-VP16-NFAT LexAop-rCD2-GFP/UAS-inx7*^{RNAi}

Mex^{TS} > + corresponds to +*; Mex-Gal4 Tubulin-Gal80*^{TS}*/UAS- emptyVK37;* + and +*; Mex-Gal4 Tubulin-Gal80*^{TS}*/ +; UAS-Luciferase*^{RNAi}*/+*

Mex^{TS} > *inx2*^{RNAi} corresponds to +*; Mex-Gal4 Tubulin-Gal80*^{TS}*; UAS-inx2*^{RNAi}/+ and +*; Mex-Gal4 Tubulin-Gal80*^{TS}*/UAS-emptyVK37; UAS-inx2*^{RNAi}*/+*

bioRxiv preprint doi: https://doi.org/10.1101/2023.08.14.553227; this version posted August 15, 2023. The copyright holder for this preprint (which was not certified by peer review) is the author/funder, who has granted bioRxiv a license to display the preprint in perpetuity. It is made available under aCC-BY 4.0 International license.

Mex^{TS} > inx2^{RNAi} + *nAChRβ3* corresponds to +*; Mex-Gal4 Tubulin-Gal80^{TS}/ UAS-nAChRβ3; UAS-inx2^{RNAi}/+*

Mex^{TS} > nAChRβ3 corresponds to +*; Mex-Gal4 Tubulin-Gal80^{TS}/* UAS-nAChRβ3;*UAS-Luciferase^{RNAi}/+*

MexLexA > LexAopGCAMP7c corresponds to +*; Mex-LexA::GAD/+; 13xLexAop-IVS-jGCaMP7c/+*

Ext. Fig. 1:

Ore R corresponds to *Oregon R*

Myo1A^{TS}> dCas9-VPR corresponds to +*; Myo1A-Gal4 Tubulin-Gal80^{TS} /+; UAS-3XFLAG-dCas9-VPR/+*

Myo1A^{TS}> dCas9-VPR, gRNA-ACE corresponds to +*; Myo1A-Gal4 Tubulin-Gal80^{TS} / gRNA-ACE; UAS-3XFLAG-dCas9-VPR/+*

Mex^{TS}> dCas9-VPR corresponds to +*; Mex-Gal4 Tubulin-Gal80^{TS}/+ ; UAS-3XFLAG-dCas9-VPR/+*

Mex^{TS}> dCas9-VPR, gRNA-ACE corresponds to +*; Mex-Gal4 Tubulin-Gal80^{TS} / gRNA-ACE; UAS-3XFLAG-dCas9-VPR/+*

How^{TS}> dCas9-VPR corresponds to +*; Tubulin-Gal80^{TS} /+; how^{24B}-Gal4/ UAS-3XFLAG-dCas9-VPR*

How^{TS}> dCas9-VPR, gRNA-ACE corresponds to +*; Tubulin-Gal80^{TS} / gRNA-ACE; how^{24B}-Gal4/ UAS-3XFLAG-dCas9-VPR*

Hml^{TS} > dCas9-VPR corresponds to +*; hml-Gal4Δ UAS-GFP/+; Tubulin-Gal80^{TS}/ UAS-3XFLAG-dCas9-VPR*

Hml^{TS} > dCas9-VPR, gRNA-ACE corresponds to +*; hml-Gal4Δ UAS-GFP// gRNA-ACE; Tubulin-Gal80^{TS}/ UAS-3XFLAG-dCas9-VPR*

Ext. Fig. 2:

Myo1A^{TS} > + corresponds to +*; Myo1A-Gal4 Tubulin-Gal80^{TS}/+; +*

Myo1A^{TS} > nAChRβ3^{RNAi} corresponds to +*; Myo1A-Gal4 Tubulin-Gal80^{TS}/+; UAS-nAChRβ3^{RNAi}/+*

Myo1A^{TS} > nAChRβ3^{RNAi-2} corresponds to +*; Myo1A-Gal4 Tubulin-Gal80^{TS}/+; UAS-nAChRβ3^{RNAi-2}/+*

Mex^{TS}> GCAMP7c corresponds to +*; Mex-Gal4 Tubulin-Gal80^{TS}/+; 20XUAS-IVS-jGCaMP7c/+*

bioRxiv preprint doi: https://doi.org/10.1101/2023.08.14.553227; this version posted August 15, 2023. The copyright holder for this preprint (which was not certified by peer review) is the author/funder, who has granted bioRxiv a license to display the preprint in perpetuity. It is made available under aCC-BY 4.0 International license.

Mex^{TS}> GCAMP7c / nAChR$\beta3^{RNAi}$ corresponds to +; Mex-Gal4 Tubulin-Gal80^{TS}/+; 20XUAS-IVS-jGCaMP7c/ UAS-nAChR$\beta3^{RNAi}$

Esg^{TS} > + corresponds to +; esg-Gal4 Tubulin-Gal80^{TS} / +; UAS- Luciferase^{RNAi}/+

Esg^{TS} > nAChR$\beta3^{RNAi}$ corresponds to +; esg-Gal4 Tubulin-Gal80^{TS}; UAS-nAChR$\beta3^{RNAi}$ /+

Su(H)GBE^{TS} > + corresponds to +; Su(H)Gbe-Gal4 UAS-CD8-GFP/+; Tubulin-Gal80^{TS}/UAS-Luciferase^{RNAi}

Su(H)GBE ^{TS} > nAChR$\beta3^{RNAi}$ corresponds to +; Su(H)Gbe-Gal4 UAS-CD8-GFP/+; Tubulin-Gal80^{TS}/ UAS-nAChR$\beta3^{RNAi}$

Pros^{TS} > + corresponds to +; Tubulin-Gal80^{TS} / +; prospero-Gal4/ UAS-Luciferase^{RNAi}

Pros^{TS} > nAChR$\beta3^{RNAi}$ corresponds to +; Tubulin-Gal80^{TS} / +; prospero-Gal4/ UAS-nAChR$\beta3^{RNAi}$

How^{TS} > + corresponds to +; Tubulin-Gal80^{TS} / +; how^{24B}-Gal4/ UAS-Luciferase ^{RNAi}

How^{TS} > nAChR$\beta3^{RNAi}$ corresponds to +; Tubulin-Gal80^{TS} / +; how^{24B}-Gal4/ UAS-nAChR$\beta3^{RNAi}$

Hml^{TS} > + corresponds to +; hml-Gal4Δ UAS-GFP/+; Tubulin-Gal80^{TS}/ UAS-Lucifersa^{RNAi}

Hml^{TS} > nAChR$\beta3^{RNAi}$ corresponds to +; hml-Gal4Δ UAS-GFP/+; Tubulin-Gal80^{TS}/ UAS-nAChR$\beta3^{RNAi}$

Mex^{TS} > + / nAChRβ3-flag corresponds to +; Mex-Gal4 Tubulin-Gal80^{TS} / nAChRβ3-flag; +

Mex^{TS} > UAS-nAChR$\beta3^{RNAi}$ / nAChRβ3-flag corresponds to +; Mex-Gal4 Tubulin-Gal80^{TS} / nAChRβ3-flag; UAS-nAChR$\beta3^{RNAi}$ /+

Ext. Fig. 3:

Myo1A^{TS} > + corresponds to +; Myo1A-Gal4 Tubulin-Gal80^{TS}/UAS-emptyVK37; +/+ and +; Myo1-Gal4 Tubulin-Gal80^{TS}/UAS-2x-GFP; +

Myo1A^{TS} > nAChR$\beta3^{RNAi}$ corresponds to +; Myo1A-Gal4 Tubulin-Gal80^{TS}/+; UAS-nAChR$\beta3^{RNAi}$/+

Myo1A^{TS} > + / Diap1-LacZ corresponds to +; Myo1A-Gal4 Tubulin-Gal80^{TS}/+; Diap1-LacZ / +

Myo1A^{TS} > nAChR$\beta3^{RNAi}$ corresponds to +; Myo1A-Gal4 Tubulin-Gal80^{TS}/+; Diap1-LacZ /UAS-nAChR$\beta3^{RNAi}$

Mex^{TS} > + corresponds to +; Mex-Gal4 Tubulin-Gal80^{TS}/UAS- emptyVK37; +

Mex^{TS} > nAChR$\beta3^{RNAi}$ corresponds to +; Mex-Gal4 Tubulin-Gal80^{TS}/+; UAS-nAChR$\beta3^{RNAi}$/+

Esg^{TS} > NFAT-CaLexA corresponds to Tubulin-Gal80^{TS}/+; esg-Gal4 / LexAop-CD8-GFP-2A-CD8GFP; UAS-mLexA-VP16-NFAT LexAop-rCD2-GFP/ +

Myo1A^{TS} > nAChR$\beta3^{RNAi-2}$ corresponds to +; Myo1A-Gal4 Tubulin-Gal80^{TS}/UAS-nAChR$\beta3^{RNAi-2}$; UAS-Luciferase/+

Myo1A^{TS}> Orai corresponds to +; Myo1A-Gal4 Tubulin-Gal80^{TS}/UAS-emptyVK37; UAS-Orai/+

bioRxiv preprint doi: https://doi.org/10.1101/2023.08.14.553227; this version posted August 15, 2023. The copyright holder for this preprint (which was not certified by peer review) is the author/funder, who has granted bioRxiv a license to display the preprint in perpetuity. It is made available under aCC-BY 4.0 International license.

Myo1A^{TS}*> nAChR*β*3*$^{RNAi-2}$ + Orai corresponds to +*; Myo1A-Gal4 Tubulin-Gal80*^{TS}*/UAS-nAChR*β*3*$^{RNAi-2}$*; UAS-Orai /+*

Myo1A^{TS}*> nAChR*β*3* corresponds to +*; Myo1A-Gal4 Tubulin-Gal80*^{TS}*/ UAS-nAChR*β*3; +*

Mex^{TS}*> GCAMP7c* corresponds to +*; Mex-Gal4 Tubulin-Gal80*^{TS}*/+; 20XUAS-IVS-jGCaMP7c/+*

Mex^{TS}*> GCAMP7c + nAChR*β*3* corresponds to +*; Mex-Gal4 Tubulin-Gal80*^{TS}*/ nAChR*β*3; 20XUAS-IVS-jGCaMP7c/ +*

<u>Ext. Fig. 4:</u>

Elav^{TS} > + corresponds to *Elav-Gal4/+; Tubulin-Gal80*^{TS}*/+; UAS-Luciferase*^{RNAi}*/+*

Elav^{TS} > *ChAT*^{RNAi} corresponds to *Elav-Gal4/+; Tubulin-Gal80*^{TS}*/+; UAS-ChAT*^{RNAi} */+*

Esg^{TS} > + corresponds to *Tubulin-Gal80*^{TS}*/+; esg-Gal4 / +; UAS- Luciferase* ^{RNAi}*/+*

Esg^{TS} > *ChAT*^{RNAi} corresponds to *Tubulin-Gal80*^{TS}*/+; esg-Gal4 / +; UAS- ChAT*^{RNAi} */+*

Pros^{TS} > + corresponds to +; *Tubulin-Gal80*^{TS} */ +; prospero-Gal4/ UAS-Luciferase*^{RNAi}

Pros^{TS} > *ChAT*^{RNAi} corresponds to +; *Tubulin-Gal80*^{TS} */ +; prospero-Gal4/ UAS-ChAT*^{RNAi}

Myo1A^{TS} > + corresponds to +; *Tubulin-Gal80*^{TS}*, myo1A-Gal4 /+ ; UAS-Luciferase* ^{RNAi}*/+*

Myo1A^{TS} > *ChAT*^{RNAi} corresponds to +; *Tubulin-Gal80*^{TS}*, myo1A-Gal4 /+; UAS-ChAT*^{RNAi}*/+*

Hml^{TS} > + corresponds to +*; hml-Gal4*Δ *UAS-GFP/+; Tubulin-Gal80*^{TS}*/ UAS-Lucifersa*^{RNAi}

Hml^{TS} > *ChAT*^{RNAi} corresponds to +*; hml-Gal4*Δ *UAS-GFP/+; Tubulin-Gal80*^{TS}*/ UAS-ChAT*^{RNAi}

How^{TS} > + corresponds to +; *Tubulin-Gal80*^{TS} */ +; how*^{24B}*-Gal4/ UAS-Luciferase* ^{RNAi}

How^{TS} > *ChAT*^{RNAi} corresponds to +; *Tubulin-Gal80*^{TS} */ +; how*^{24B}*-Gal4/ UAS-ChAT*^{RNAi}

Esg > + corresponds to +; *esg-Gal4 / UAS-emptyVK37; +*

Esg > *sc*^{RNAi} corresponds to +; *esg-Gal4/+; sc*^{RNAi} */+*

ChAT > *mCD8GFP* corresponds to +*; 10xUAS-mCD8::GFP/+ ; ChAT*^{MI04508}*-Gal4/* + and +*; +; ChAT*^{MI04508}*-Gal4/ 10xUAS-mCD8::GFP*

ChAT > *6XGFP* corresponds to +*; +; ChAT*^{MI04508}*-Gal4/ 20XUAS-6XGFP*

ChAT-QF> QUAS-TomatoHA+myo1A > GFP corresponds to *UAS-mCD8::GFP, QUAS-mtdTomato-3xHA/+; Myo1A-Gal4, UAS-GFP/ + ; ChAT-QF/+*

ChAT> mCD8GFP/ Tsh-Gal80 corresponds to +*; 10xUAS-mCD8::GFP/ Tsh-Gal80 ; ChAT*^{MI04508}*-Gal4/ +*

R49E06> mCD8GFP corresponds to +*; 10xUAS-mCD8::GFP/+; R49E06-Gal4/+*

R49E06> 2xGFP corresponds to +*; UAS-2x-GFP/+; R49E06-Gal4/+*

<u>Ext. Fig. 5:</u>

bioRxiv preprint doi: https://doi.org/10.1101/2023.08.14.553227; this version posted August 15, 2023. The copyright holder for this preprint (which was not certified by peer review) is the author/funder, who has granted bioRxiv a license to display the preprint in perpetuity. It is made available under aCC-BY 4.0 International license.

R49E06^{TS} *> syt1HA* corresponds to +*; Tubulin-Gal80*^{TS}*/* +; *R49E06-Gal4/5xUAS-IVS-Syt1::smGdP-HA*

R49E06-QF> QUAS-TomatoHA corresponds to +*; +; R49E06-QF/ QUAS-mtdTomato-3xHA*

mexLexA> LexAopGFP corresponds to +*; 13xLexAop2-sfGFP/+; mex-LexA::GAD/ +*

mexLexA^{TS}*> LexAopnAChRβ3* corresponds to +*; Tubulin-Gal80*^{TS}*/ LexAop-nAChRβ3*; *mex-LexA::GAD / +*

R49E06^{TS}*> syt1HA + mexLexA > LexAop6xGFP* corresponds to +; *Tubulin-Gal80*^{TS}*/ 13xLexAop2-6xGFP; R49E06-Gal4 mex-LexA::GAD/ 5xUAS-IVS-Syt1::smGdP-HA*

R49E06^{TS}*> syt1HA + mexLexA > LexAopxGFP* corresponds to +; *Tubulin-Gal80*^{TS}*/ 13xLexAop2-sfGFP; R49E06-Gal4 mex-LexA::GAD/ 5xUAS-IVS-Syt1::smGdP-HA*

R49E06^{TS}*>* + corresponds to +*; Tubulin-Gal80*^{TS}*/+; R49E06-Gal4; UAS-Luciferase*^{RNAi}

R49E06^{TS}*> ChAT*^{RNAi} corresponds to +*; Tubulin-Gal80*^{TS}*/+; R49E06-Gal4; UAS-ChAT*^{RNAi}

ARCENs>+ corresponds to +*; UAS-2xGFP/+; R49E06-Gal4/+*

ARCENs> TrpA1 corresponds to +*; UAS-TrpA1/+; R49E06-Gal4/+*

ARCENs> TrpA1/ChATGal80 corresponds to +*; UAS-TrpA1/+; R49E06-Gal4/ChAT-Gal80*

ARCENs^{TS}*>* + corresponds to +*; Tubulin-Gal80*^{TS}*/+; R49E06-Gal4; UAS-Luciferase*^{RNAi}

ARCENs^{TS}*> wgn*^{RNAi} corresponds to +*; Tubulin-Gal80*^{TS}*/UAS-wgn*^{RNAi}*; R49E06-Gal4/+*

ARCENs^{TS}*> grnd*^{RNAi} corresponds to +*; Tubulin-Gal80*^{TS}*/UAS-grnd*^{RNAi}*; R49E06-Gal4/+*

Ext. Fig. 6:

Mex^{TS} > + corresponds to +*; Mex-Gal4 Tubulin-Gal80*^{TS}*/UAS- emptyVK37; +*

Mex^{TS} *> inx2*^{RNAi} corresponds to +*; Mex-Gal4 Tubulin-Gal80*^{TS}*; UAS-inx2*^{RNAi}*/+*

Mex^{TS} *> inx7*^{RNAi} corresponds to +*; Mex-Gal4 Tubulin-Gal80*^{TS}*; UAS-inx7*^{RNAi}*/+*

Supplementary Txt

gBlock1:

AACGAGGATTATCATCAAAAGAGCGCCGGAGTATAAGTAGAGGCGCTTCGTCTACGGAGC
GACAATTCAATTCAAACAAGCAAAGTGAACACGTCGCTAAGCGAAAGCTAAGCAAATAAAC
AAGCGCAGCTGAACAAGCTAAACAATCTGCAGTAAAGTGCAAGTTAAAGTGAATCAATTAA
AAGTAACCAGCAACCAAGTAAATCAACTGCAACTACTGAAATCTGCCAAGAAGTAATTATTG
AATACAAGAAGAGAACTCTGAATACTTTCAACAAGTTACCGAGAAAGAAGAACTCACACACA
GCGGCCAATTCGGTACCGCGGCCGCTAAGCAA

bioRxiv preprint doi: https://doi.org/10.1101/2023.08.14.553227; this version posted August 15, 2023. The copyright holder for this preprint (which was not certified by peer review) is the author/funder, who has granted bioRxiv a license to display the preprint in perpetuity. It is made available under aCC-BY 4.0 International license.

gBlock2:

ATATTTTTTATATACATACTTTTCAAATCGCGCGCCCTCTTCATAATTCACCTCCACCACACC
ACGTTTCGTAGTTGCTCTTTCGCTGTCTCCCACCCGCTCTCCGCAACACATTCACCTTTTGT
TCGACGACCTTGGAGCGACTGTCGTTAGTTCCGCGCGATTCGGTTCGCTCAAATGGTTCC
GAGTGGTTCATTTCGTCTCAATAGAAATTAGTAATAAATATTTGTATGTACAATTTATTTGCT
CCAATATATTTGTATATATTTCCCTCACAGCTATATTTATTCTAATTTAATATTATGACTTTTTA
AGGTAATTTTTTGTGACCTGTTCGGAGTGATTAGCGTTACAATTTGAACTGAAAGTGACATC
CAGTGTTTGTTCCTTGTGTAGATGCATCTCAAAAAAATGGTGGGCATAATAGTGTTGTTTAT
ATATATCAAAAATAACAACTATAATAATAAGAATACATTTAATTTAGAAAATGCTTGGATTTCA
CTGGAACTGCTACACCGATCACTTAGGACCCATAATGCATCATTGGAGGTGAAGAAAATCT
GCTGAATGTTCTTCGATGTGATATATATGATTTCGTTGCCCTCGGCTGTGATGTTGGCTAGT
TTGGCACATCGTACGACAGCTCCGAATTTGGGGATAACATAGTACAATGTTCCGAAATTGT
CGATGATCATGCTGCGACTGCTGCCCAGAAGACTGCCCAAATGTGTGAGTTTAATGCTATT
GTTAATTGGTTTGCTTGCTAATTCGCTTTTGCCTTCAATTTCCAGTCTGTCCACCGTACTGT
ATAGATCACCATCCTGGTCGGAGAGTATGAGTTCCCCTTGAATTCCGAAGATGAAATCCAC
TGGCTTGATGGGAAACGATTGATTCATATTCTCGTACTTATTGCTCTCCAAGGATAGGCGG
TGCCACGTCTGCTCCAGGATGTCGTAGGCTAGAATTTCTGGTACCTTTCCAAGGATGAAGT
AAATATGGCGCTCGTGCTCGCATCCCGGACTCTTGGGTCCCATTTGGACAGTCAGAAAGT
GGGTGTCATTAGCACCTATATGGTGTCCACAATCGATTCTCAATAGCTCCGCGTTGTTACG
CATCAGATCGAAGACAAACAAACGGGGTGAGCACGTGCTGCCGGGAAATCCGATGTCCAT
GACCCACAGTCGACTCAGTGCATCCACCTGGGACCAGTGTGCCTGTTGTACCAGAGAGCA
ATCATCATGATCCCCCATGGAATGAACATCGATGTGGGGAAAGACGACAGTGGGCATGGG
AAAATAGGAAACCGGCCATTGCGCCTCGATCAAAGTGGGCGCATCGTTGCTTTCTACATTT
ACGCTGAGAAACACTCGCGAAAAGTGCATCGATAGATGACGGACTTTGTAGCTGGATTCGT
TTAAACCAGATTCGGATAACAGCCAGGCGTCCGTGGCCAGAATTAGACCAACAATGAGAAA
TCCCAAGCCGAGGACACCATGTGTGACCATCTTTAAACGCGAATTCAGACTGAGCTTAGGG
CTAGAGAACTCTGCTTACGTTGTAAGTGCCCGTTCAAGAAGTCCACCAAAGTGTCTACATC
AGCAGTTGGGATCGGGTGTCGCCGAATATATCGGCGACCTGCATTAATGGGGACCAGCAT
TGGAGTGTTTCTCTATTCACTTGTGGTCCCAAAAAAAAAAAAGCTTACCAATTTCAATCGGAG
AATATCGAAATCATGACACAGTTTCCTCTTAAAATTACCCAAAATTGGATTGTTATATTTTTC
AAAATAAATCAAAGTTAAAACCAACAAAATGGTTGTTTTGTACCACCATAATAACCTATAATA
AATAAATCATAATAAGGCTATTTTTGTCGAACTTTAGCGATATTGATAAGGTTCCTGTGAACT
GAACCTACCTTAAGTGCTTAGTTTTAACATAACCTTAATGTTAACGAGTATTTCTTCGCGTAA
AAGTACAAAAAAACCCTATCATATCTTTACAGCCATTATAAAGATTGCTTATAGATGACTGAT

bioRxiv preprint doi: https://doi.org/10.1101/2023.08.14.553227; this version posted August 15, 2023. The copyright holder for this preprint (which was not certified by peer review) is the author/funder, who has granted bioRxiv a license to display the preprint in perpetuity. It is made available under aCC-BY 4.0 International license.

TTCACTTGGGAATTGCCAAAAAGCTAAAGTGAACCCCCGAGGAATCAATTAATGTTGTTTAG
CTTTCAGTTTTTAGTACATTCGTATTTTTTTAAGTATTAAAATTATTTAATCACACGAATAAAC
ATTAAGAAATATTTTATTCTGGAAACTTTCTTCGTTATAGAGCATTTATCGTTGTAAAAAAAAT
AATTTCATTAGTTTACATTACATTTTCGTTTATATGTTTAGATTTTTTCTAATTAAATGATATTG
TATAATTTATTGCAAAAATTATAATACTCACTTCAAATAATCTGAAACAAATCCACCCACTCC
GAAAAATGTGGTAAGCTTTATTTTCTAACACCTCTCTTCTGATTTTATTGATTATAACTGGAA
TCTTTATCGGTTTCTCTCTTTTTCCATCAGTAGGTTGAGAGCTTTTCATACTTTTCACGCCGA
AATGGGAAAAATGGCACTGCGCGTAGAGTCAGTTATCCATTTGAGTTGGCCAAGATCAGAT
CGAAATACGCTAAAATCATTTTCGGGAGCACAATCGAATACTCATACAACACACACTTCAAA
CGAGGATTATCATCAAAAGAGCGCCGG
